# Genetic Insights and Diagnostic Challenges in Highly Attenuated Lysosomal Storage Disorders

**DOI:** 10.3390/genes16080915

**Published:** 2025-07-30

**Authors:** Elena Urizar, Eamon P. McCarron, Chaitanya Gadepalli, Andrew Bentley, Peter Woolfson, Siying Lin, Christos Iosifidis, Andrew C. Browning, John Bassett, Udara D. Senarathne, Neluwa-Liyanage R. Indika, Heather J. Church, James A. Cooper, Jorge Menendez Lorenzo, Maria Elena Farrugia, Simon A. Jones, Graeme C. Black, Karolina M. Stepien

**Affiliations:** 1 Adult Inherited Metabolic Diseases, Salford Royal Hospital, Northern Care Alliance NHS Foundation Trust, Salford M6 8HD, UK; elena.urizar@scsalud.es (E.U.); e.p.mccarron@doctors.org.uk (E.P.M.); john.bassett@nca.nhs.uk (J.B.); Jorge.menendezlorenzo@nhs.scot (J.M.L.); 2 Ear Nose and Throat Surgery, Salford Royal Hospital, Northern Care Alliance NHS Foundation Trust, Salford M6 8HD, UK; 3 Respiratory Medicine Department, Wythenshawe Hospital, Manchester University NHS Foundation Trust, Manchester M23 9LT, UK; andrew.bentley@mft.nhs.uk; 4 Manchester Academic Health Sciences Centre, University of Manchester, Manchester M13 9WL, UK; 5 Cardiology Department, Salford Royal Hospital, Northern Care Alliance NHS Foundation Trust, Salford M6 8HD, UK; peter.woolfson@nca.nhs.uk; 6 Division of Evolution, Infection and Genomics, School of Biological Sciences, Faculty of Biology, Medicine and Health, University of Manchester, Manchester M13 9PL, UK; siying.lin@mft.nhs.uk; 7 Manchester Royal Eye Hospital, Manchester University NHS Foundation Trust, Manchester M13 9WL, UK; christos.iosifidis@mft.nhs.uk (C.I.); graeme.black@mft.nhs.uk (G.C.B.); 8 Newcastle Eye Centre, Royal Victoria Infirmary, Newcastle upon Tyne NE1 4LP, UK; andrew.browning@nhs.net; 9 Department of Biochemistry, Faculty of Medical Sciences, University of Sri Jayewardenepura, Nugegoda 10250, Sri Lanka; udara.senarathne@sjp.ac.lk (U.D.S.); ind.liyanage@sjp.ac.lk (N.-L.R.I.); 10 Department of Medicine, School of Clinical Sciences at Monash Health, Monash University, Melbourne, VIC 3168, Australia; 11 Willink Biochemical Genetics Laboratory, St Mary’s Hospital, Manchester University NHS Foundation Trust, Manchester M13 9WL, UK; heather.church@mft.nhs.uk (H.J.C.); james.cooper@mft.nhs.uk (J.A.C.); 12 Institute of Neurological Sciences, Queen Elizabeth University Hospital, Glasgow G51 4TF, UK; mariaelena.farrugia2@nhs.scot; 13 Genomic Medicine, Manchester University NHS Foundation Trust, Manchester M13 9WL, UK; simon.jones@mft.nhs.uk; 14 Division of Cardiovascular Sciences, University of Manchester, Manchester M13 9PL, UK

**Keywords:** lysosomal storage diseases, attenuated form, enzyme activity

## Abstract

Background: Lysosomal storage diseases (LSDs) are a genetically and clinically heterogeneous group of inborn errors of metabolism caused by variants in genes encoding lysosomal hydrolases, membrane proteins, activator proteins, or transporters. These disease-causing variants lead to enzymatic deficiencies and the progressive accumulation of undegraded substrates within lysosomes, disrupting cellular function across multiple organ systems. While classical phenotypes typically manifest in infancy or early childhood with severe multisystem involvement, a combination of advances in molecular diagnostics [particularly next-generation sequencing (NGS)] and improved understanding of disease heterogeneity have enabled the identification of attenuated forms characterised by residual enzyme activity and later-onset presentations. These milder phenotypes often evade early recognition due to nonspecific or isolated symptoms, resulting in significant diagnostic delays and missed therapeutic opportunities. Objectives/Methods: This study characterises the clinical, biochemical, and molecular profiles of 10 adult patients diagnosed with LSDs, all representing attenuated forms, and discusses them alongside a narrative review. Results: Enzyme activity, molecular data, and phenotypic assessments are described to explore genotype–phenotype correlations and identify diagnostic challenges. Conclusions: These findings highlight the variable expressivity and organ involvement of attenuated LSDs and reinforce the importance of maintaining clinical suspicion in adults presenting with unexplained cardiovascular, neurological, ophthalmological, or musculoskeletal findings. Enhanced recognition of atypical presentations is critical to facilitate earlier diagnosis, guide management, and enable cascade testing for at-risk family members.

## 1. Introduction

Lysosomal storage disorders (LSDs) encompass a group of over 50 inherited metabolic conditions resulting from pathogenic variants in genes encoding lysosomal hydrolases or associated proteins essential for macromolecule degradation and cellular recycling processes [1]. These deficiencies lead to the pathological accumulation of undegraded substrates within lysosomes, thereby disrupting multiple cellular pathways including intracellular trafficking, autophagic flux, mitochondrial integrity, calcium signalling, and immune responses [2]. Although individually rare, collectively LSDs have an estimated incidence of 1 in 5000 live births [3,4].

Traditionally, LSDs have been described in the context of severe, early-onset, multisystem presentations. However, with the broader adoption of expanded newborn screening (NBS) and increasing use of next-generation sequencing (NGS) in atypical or previously undiagnosed cases, the phenotypic spectrum of LSDs has significantly widened [5]. These technological advances have facilitated the identification of milder or late-onset forms, typically characterised by residual enzyme activity and a more indolent, non-classical course [6]. These variants commonly present during adolescence or adulthood, often manifesting with subtle, isolated, or nonspecific findings, with some predominantly affecting single organ systems such as the heart, joints, eyes, or the central nervous system.

One of the major challenges in diagnosing attenuated LSDs is the substantial phenotypic overlap with both common acquired conditions and other monogenic disorders affecting overlapping single organ systems. Moreover, standard enzymatic assays may yield borderline or inconclusive results, especially in the context of pseudodeficiency alleles (genetic variants associated with reduced enzyme activity but without clinical disease) further complicating diagnosis [7]. In addition, routine biochemical markers such as urine glycosaminoglycans (uGAGs), may also lack sufficient sensitivity in these presentations [8], and as a result new diagnostic techniques have been developed in quantification of urinary GAGs by using tandem mass spectrometry [9,10]. Correlation between residual enzyme activity, age of onset, and disease severity has been well established, with attenuated phenotypes tending to have later and more variable symptom onset [11]. Due to subtle clinical features, patients frequently undergo evaluation by multiple specialties including neurology, cardiology, rheumatology, ophthalmology, or psychiatry before a unifying diagnosis is considered. Consequently, misdiagnoses and diagnostic delays are common, often resulting in irreversible organ damage. Doerr et al. [12] described a prolonged “diagnostic odyssey” spanning up to a decade from symptom onset to definitive diagnosis, frequently experiencing psychological distress and missed therapeutic opportunities. A global survey of adult metabolic centres further indicated that attenuated LSDs represent an increasingly prevalent metabolic diagnosis among adults [13]. Although access to molecular diagnostics has increased diagnostic yield, early recognition still heavily depends on clinical suspicion. Molecular testing also presents challenges when variants of unknown significance are found, making clinical correlation ever more important. This is especially pertinent in adult patients presenting with unexplained symptoms or slowly progressive single organ or multisystem involvement. Thus, increased awareness and multidisciplinary collaboration remain essential to optimise outcomes in this evolving patient population [6,14].

The aim of this study is to characterise the clinical, biochemical, and molecular features of attenuated LSDs diagnosed in adolescence or adulthood, using data from a cohort of 10 patients across the LSD spectrum, to gain insights into diagnostic patterns, highlight common pitfalls, and illustrate phenotypic diversity. Our findings are presented alongside a narrative review to inform targeted diagnostic strategies and raise clinical awareness of these presentations.

## 2. Materials and Methods

This study retrospectively reviewed 10 adult patients diagnosed with attenuated forms of LSDs, encompassing a range of conditions. Some of the included patients have been previously described in larger case series or individual published reports (see * in Table 1). Data were extracted from clinical records, laboratory results, and molecular diagnostic reports. Additionally, patient findings were contextualised through comparison with the relevant literature as part of a narrative review.

All patients had previously provided informed consent for publication. In accordance with institutional policy, ethical approval was not required for the analysis and presentation of fully anonymized retrospective data. Institutional standards for anonymity and data protection guidelines were adhered to throughout the study, ensuring compliance with relevant local governance standards.

## 3. Results (Overview)

Table 1 summarises the clinical and biochemical characteristics of 10 adult patients diagnosed with attenuated LSDs, including mucopolysaccharidosis (MPS) types I, II, IIIA, IVA, and VI, Alpha-/Beta-mannosidosis, Krabbe disease, and multiple sulfatase deficiency (MSD). The median age at diagnosis was 43 years (range 8–56). Enzyme activity was reduced (to varying degrees) or deficient in all cases, confirming diagnosis. Cardiac manifestations included left ventricular hypertrophy (LVH), valvular pathology (aortic stenosis [AS]), and arrhythmias such as atrial fibrillation (AF). Ear, nose, and throat (ENT) and respiratory findings, including obstructive sleep apnoea (OSA), hearing impairment, bulky upper airways, and central airway narrowing, were frequently observed. Hepatosplenomegaly was mild or absent in most cases. Musculoskeletal features varied from joint restriction to skeletal dysplasia and scoliosis. Neurological manifestations included intellectual disability (ID), ataxia, peripheral neuropathy, and cerebral white matter changes. Ophthalmologic involvement such as retinal degeneration and corneal involvement was documented in several patients, with notably prominent ocular involvement in a patient with an MPS IIIA variant. The clinical, biochemical, and molecular findings described in detail below illustrate the heterogeneity of these LSD phenotypes and aim to guide diagnostic recognition in adult populations.

## 4. Clinical Manifestations

LSDs encompass a broad spectrum of phenotypes. In attenuated forms, disease progression is slower, and organ involvement may be subtler, often delaying diagnosis, as reflected in our adult patient cohort. The following section provides a system-by-system review of the literature, highlighting key clinical features of LSDs diagnosed in adulthood, complemented by findings from our patient series. Anthropometric measurements revealed some patients had short stature. Almost all MPS and Alpha-mannosidosis patients had restricted neck extension of some degree.

### 4.1. Cardiac Manifestations

**Figure 1:**
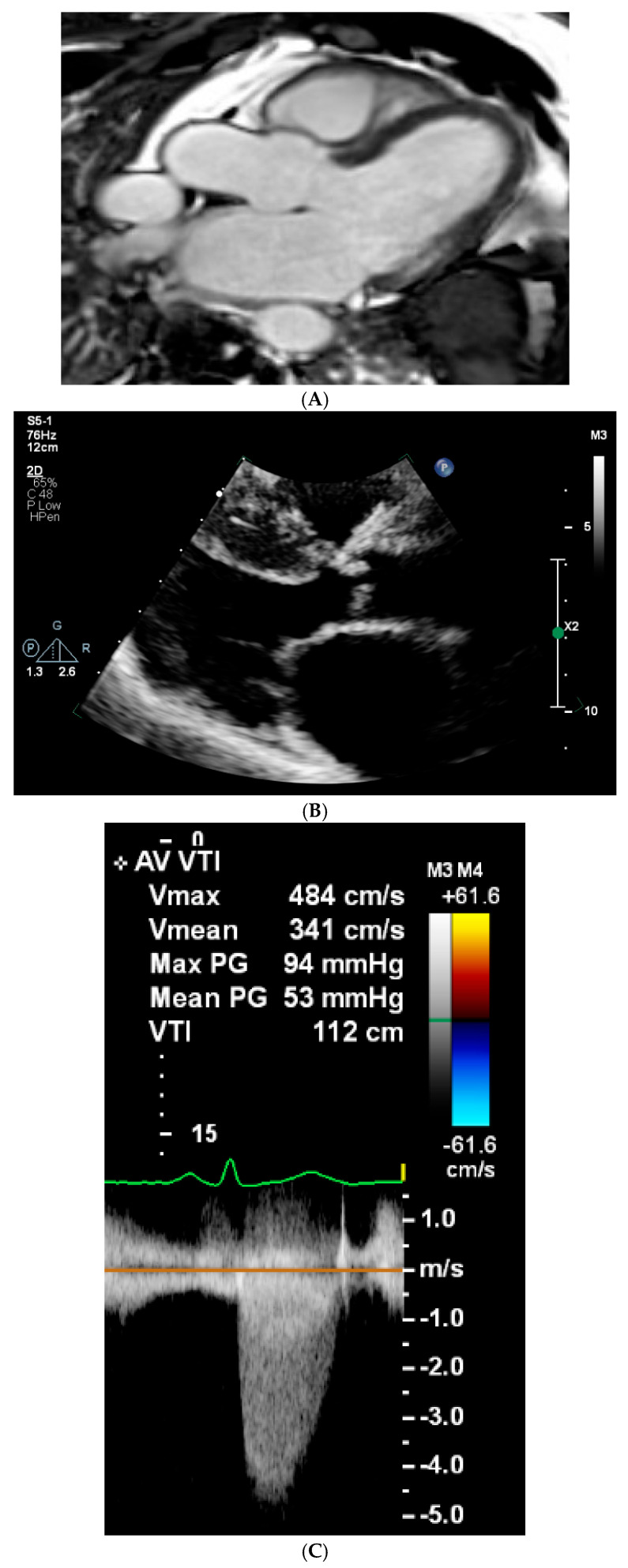
Cardiac manifestations in patients with attenuated MPS II (**A**) and MPS VI (**B**,**C**). (**A**) Cardiac magnetic resonance imaging (MRI) in a patient with attenuated MPS II demonstrates focal myocardial fibrosis in the basal to mid-lateral wall of the left ventricle. Fibrotic changes are consistent with glycosaminoglycan (GAG) accumulation and support subclinical myocardial involvement despite minimal valvular abnormalities. (**B**) A transthoracic echocardiogram (parasternal long axis (PLAX) view) in a patient with MPS VI demonstrating GAG-related infiltration of the aortic valve and severe aortic stenosis. This PLAX view shows marked thickening and restricted mobility of the aortic valve leaflets, consistent with severe aortic stenosis. The echocardiographic findings reflect GAG accumulation within valvular tissue, a recognised cardiac manifestation in MPS VI. (**C**) A transthoracic echocardiogram (continuous-wave Doppler) in a patient with MPS VI demonstrating haemodynamically severe aortic stenosis. Continuous-wave Doppler tracing across the aortic valve shows a peak velocity (Vmax) of 4.84 m/s and a mean pressure gradient of 53 mmHg, with a peak gradient of 94 mmHg. The velocity time integral (VTI) is 112 cm. These values are consistent with severe aortic stenosis and reflect the functional consequence of GAG infiltration of the valve apparatus.

Cardiac involvement is a hallmark of several LSDs. In attenuated forms, cardiac manifestations are typically milder and present later in life. Valvular thickening, predominantly affecting the mitral and aortic valves, is common in MPS I, II, and VI, potentially leading to valvular insufficiency, stenosis, and subsequent LVH, or pulmonary hypertension [15,16,17]. This pattern was evident in our cohort, where echocardiographic evaluations revealed AS (Cases 2 and 6) (Figure 1A–C) and LVH (Case 6) in patient with MPS II and MPS VI (Figure 1A,B). Notably, isolated LVH without significant valve involvement has also been described in MPS VI [18].

Rarely, patients present with heart failure as the initial symptom [19]. Vascular abnormalities, including intima-media thickening and increased arterial stiffness, have been observed, especially in MPS VI [18,20]. Arrhythmias such as AF are also reported and may require Holter monitoring for detection [17,21,22]. In our series, AF was present in a patient with MPS II (Case 2) while T wave abnormalities were observed in patients with MPS I (Case 1).

### 4.2. Neurology Manifestations

Neurological involvement is heterogeneous. Adult-onset MLD (metochromatic leukodystrophy) and Krabbe disease may present with progressive spastic paraparesis, ataxia, or peripheral neuropathy [23,24,25]. This aligns with findings in our patient with Krabbe disease (Case 9), who demonstrated ataxia and characteristic corticospinal tract signal changes on magnetic resonance imaging (MRI). Cerebellar signs and parkinsonism can occur in GM1 and GM2 gangliosidosis [26,27], while Alpha-mannosidosis may manifest with broad-based ataxia and tremor [28]. In Fabry disease, neuropathic symptoms such as acroparesthesia may be presented even without classic features [29]. The skeletal abnormalities, including kyphoscoliotic changes and accelerated mechanical degenerative changes in the spine, may cause spinal cord and multilevel root abnormalities. There may be an element of traction on the cord and roots, or compression, with resulting mobility difficulties secondary to this (as seen in Case 4 with MPS IV).

Cognitive decline in milder forms of MPS IIIA and IIIB is typically stable or slowly progressive [30,31,32]. Notably, Case 3 with MPS IIIA demonstrated late-onset neurocognitive regression, including acute psychosis and sleep disturbances. Milder intellectual impairment has been reported noted in MPS I and II, as well as Alpha-mannosidosis [33], which is consistent with the moderate intellectual disability observed in our cohort. Psychiatric manifestations, including psychosis, hallucinations, and emotional dysregulation, have been recognised in patients with attenuated MPS III, Niemann–Pick type C (NPC), and MLD [34,35], and become pronounced later in their life.

### 4.3. Dental and Craniofacial Abnormalities

Dental crowding, malocclusion, and hypodontia are frequently observed in milder forms of MPS I and III, often in the absence of the coarsened facial features associated with classical forms [20]. Gingival hyperplasia, a characteristic finding in infantile GM1 gangliosidosis, is usually absent in adult-onset forms [36]. In our cohort, maxillary hypoplasia and poor dental hygiene were noted in cases with MPS IVA and VI, consistent with mild craniofacial dysmorphism.

### 4.4. ENT and Respiratory Manifestations

**Figure 2:**
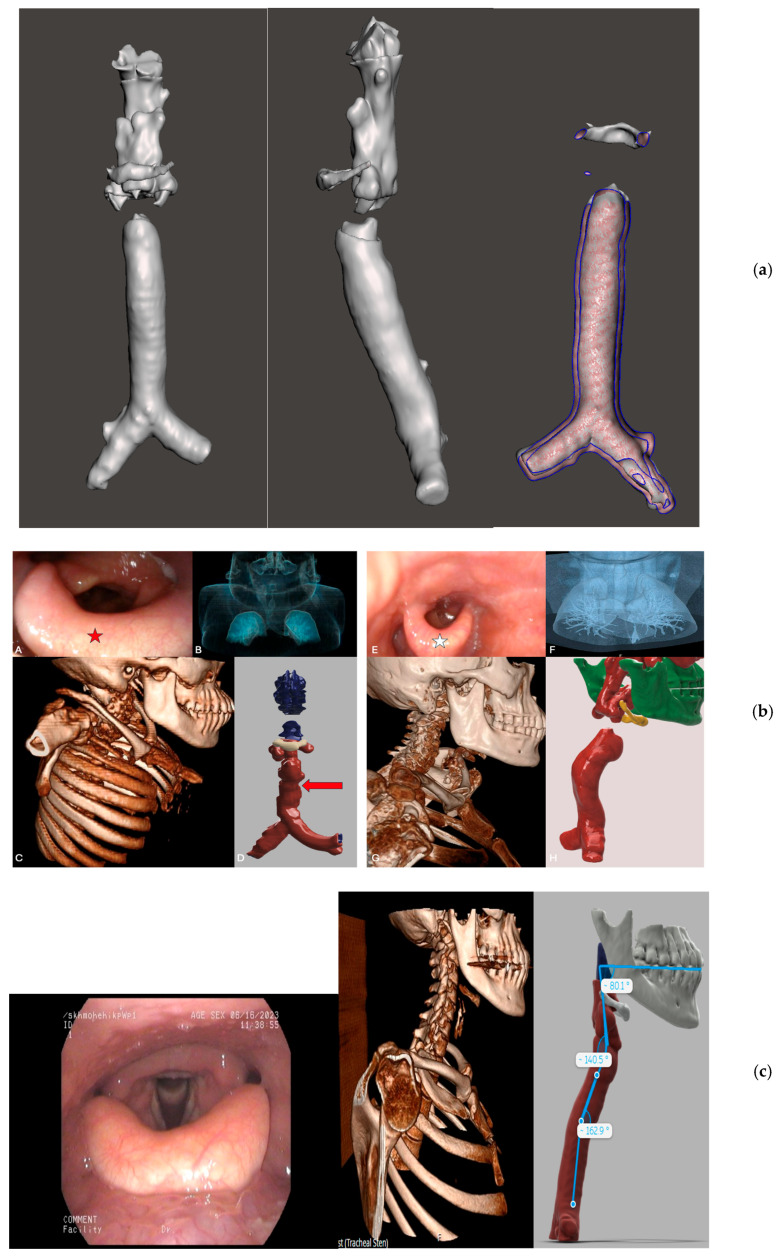
Airway and craniofacial imaging findings in patients with mucopolysaccharidoses (MPS): anatomical variation across phenotypic severity and subtypes: MPS II (**a**), IV (**b**), and VI (**b**). (**a**) A three-dimensional computed tomography (CT) reconstruction of the airway in a patient with MPS II showing tracheobronchial narrowing. The volume-rendered 3D reconstruction reveals a mildly curved trachea with irregular narrowing of the right main bronchus. These structural changes are consistent with glycosaminoglycan (GAG) deposition and airway remodelling. Although subtle, such abnormalities may predispose to ventilation asymmetry, recurrent infections, and perioperative risk. Three-dimensional airway imaging provides valuable spatial information that may not be fully appreciated on axial slices alone and is particularly useful in surgical or anaesthetic planning. (**b**) Upper airway and thoracic imaging in patients with MPS IVA, comparing classical and attenuated phenotypes. Panels (**A**–**D**) show findings from a patient with the classical MPS IVA phenotype. Nasal endoscopy (**A**) reveals a large epiglottis and markedly bulky supraglottis (red star). CT scout reconstructions (**B**,**C**) demonstrate disproportionate skeletal development, with a large head, small torso, and multiple sites of tracheal narrowing (red arrows). Three-dimensional CT reconstruction (**D**) further illustrates diffuse airway irregularity and reduced thoracic volume relative to cranial size, consistent with severe skeletal dysplasia and airway compromise. Panels E–H depict a patient with an attenuated MPS IVA phenotype. Nasal endoscopy (**E**) shows a moderately bulky supraglottis (white star) and a high anteriorly placed larynx. CT scout views (**F**,**G**) reveal milder skeletal abnormalities involving the spine and chest. Three-dimensional airway reconstruction (**H**) shows a curved but non-tortuous trachea with no significant focal narrowing. This comparison highlights the variable airway and skeletal involvement across the MPS IVA spectrum and the value of multimodal imaging in phenotype stratification. (**c**) Upper airway and skeletal imaging in a patient with MPS VI. Left: Nasal endoscopy demonstrates a large epiglottis and mildly bulky supraglottis with an anteriorly positioned larynx. Middle: CT scout imaging shows a short cervical spine, a large protruding mandible, and reduced neck extension, all contributing to altered upper airway alignment. Right: Three-dimensional CT reconstruction quantifies the oropharyngeal angulation and tracheal tilt, with the hyoid bone positioned below the mandibular plane. These structural changes may complicate airway management and highlight the need for anticipatory planning in patients with skeletal and soft tissue involvement.

Obstructive sleep apnoea (OSA) is prevalent in MPS and Mucolipidosis (ML) due to upper airway narrowing caused by GAG accumulation, adenotonsillar hypertrophy, and structural deformities [37,38]. In our cohort, adenoid hypertrophy and narrowed upper airways were reported in patients with MPS IVA (Case 4) and MPS VI (Case 6) (Figure 2b,c), while ENT abnormalities, such as an enlarged tongue and supraglottis, were frequent across several cases, including Alpha-mannosidosis (Case 7). Delayed recognition of OSA may be related to the nonspecific nature of symptoms of hypersomnolence, morning headaches, and snoring. The mainstay of treatment is continuous positive airways pressure (CPAP) or non-invasive ventilation (NIV) if there is evidence of associated hypercapnia. Compared with classical LSDs, the severity of OSA in attenuated forms is more commonly less severe, in part due to the presence of residual enzyme activity and less substrate deposition, and the use of CPAP and NIV is less frequent.

Tracheobronchial narrowing is a well-documented feature in MPS types I, II, IV, and VI, although symptoms may be subtle [6]. Imaging in our cohort revealed mild to moderate airway compromise in patients with MPS II and MPS IVA (Figure 2a,b), narrowing of the right main bronchus (MPS II), curved lower trachea (MPS IVA), and flattening of trachea (MPS IVA, Alpha-mannosidosis). Awareness of the development of tracheobronchomalacia (TBM) is important at least in part because of impaired secretion clearance and development of recurrent chest infections. In addition to airway narrowing seen on inspiratory and expiratory cross sectional CT images, recurrent infections can, over time, lead to bronchial wall thickening and bronchiectasis. The delayed onset and milder disease reduce the risk of developing more severe respiratory complications.

Additionally, restrictive lung disease and secondary pulmonary hypertension have been described in MPS VI and ML III [15], and can be related to both the underlying lung disease and skeletal chest wall deformity, leading to chronic hypoxia and ultimately type 2 respiratory failure. Interstitial lung disease has been reported in some lysosomal storage disorders, including Acid Sphingomyelinase Deficiency (ASMD) and, more rarely, in Gaucher disease—typically in neuronopathic subtypes or in patients with a history of splenectomy and significant disease burden. This highlights that attenuated LSDs can be associated with obstructive, restrictive, and mixed lung function defects.

Hearing loss, either sensorineural or conductive, is common in MPS I, II, IVA, and Alpha-mannosidosis, often progressive and predominantly affecting high-frequency hearing [15,39]. In our cohort we have noted early-onset sensorineural hearing loss (MPS I, II, III, VI, Alpha/Beta-mannosidosis,) and conductive hearing loss (MPS IVA).

### 4.5. Hepatic and Splenic Manifestations

Organomegaly is generally absent or mild (Gaucher disease and ASMD). In our cohort, hepatosplenomegaly was variably present, with splenomegaly and fatty liver noted in MPS IVA (Case 5). In contrast, Case 7 with Alpha-mannosidosis exhibited no overt organ involvement, consistent with the existing literature indicating that hepatosplenomegaly is less frequently observed in adult-onset Alpha-mannosidosis (and GM1 gangliosidosis) [40,41,42]. Late-onset lysosomal acid lipase (LAL) deficiency may feature hepatic dysfunction without adrenal calcifications (typically seen in the Wolman disease phenotype) [43].

### 4.6. Musculoskeletal Manifestations

Milder phenotypes often feature joint stiffness, scoliosis, or reduced range of motion without the severe dysostosis seen in classical forms of MPS. In our cohort, patients with MPS IVA (Cases 4 and 5) exhibited skeletal deformities, hip dysplasia, and fractures, with one individual showing significant mobility decline by age 8 (Case 4). Carpal tunnel syndrome and cervical myelopathy were frequent in MPS I and II, aligning with the literature [44,45,46,47,48,49,50,51,52]. Interestingly, in this patient group, diagnosis of carpal tunnel syndrome preceded diagnosis of MPS I [44].

Although Gaucher disease was not represented in this series, its skeletal manifestations including bone pain, osteopenia, and avascular necrosis share overlapping features with other LSDs [29,53]. Krabbe disease may also manifest with scoliosis and pes cavus [54]. Notably, gross skeletal dysmorphism (dysostosis multiplex) is not observed in Krabbe disease [6,55].

### 4.7. Cutaneous Features

Cutaneous manifestations are often reduced or absent. In Fabry disease, angiokeratomas, a hallmark feature, may be sparse or missing entirely in late-onset phenotype [29]. Similarly, the characteristic Mongolian spots or gingival hypertrophy, commonly seen in infantile GM1 gangliosidosis may not be evident [56].

### 4.8. Ophthalmic Manifestations

Retinitis pigmentosa is an atypical manifestation observed in patients with attenuated MPS II [57], MPS IIIA [32], and MPS IIIC [58]. Corneal clouding is a hallmark of MPS types I, IV, and VI, although it may be subtle in attenuated cases [59,60]. Retinopathy, including pigmentary changes and progressive vision loss, may occur in MPS I, II, IIIA, and IIIC as the predominant extra-CNS manifestation, even in the absence of other systemic features [21,61,62,63] (Table 1). A shared distinctive electroretinographic feature is a consistently reduced b:a ratio and/or an electronegative waveform; this is thought to reflect residual activity from the dark-adapted cone photoreceptor system, becoming more apparent when rod photoreceptor function is severely impaired [61,62,64,65,66].

**Figure 3:**
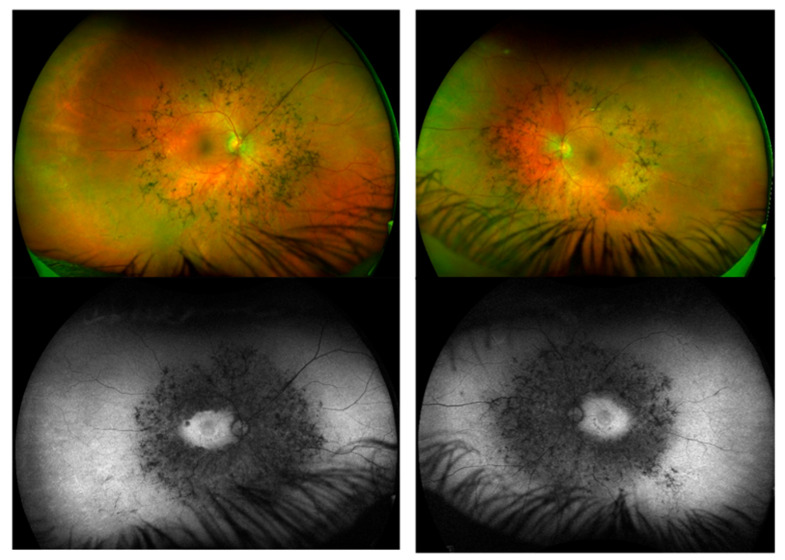
Ophthalmic manifestations in attenuated MPS I. MPS I retinal pseudocolour and fundus autofluorescence images for a 56-year-old patient with an attenuated MPS I phenotype, demonstrating pericentral retinitis pigmentosa; the retinas show an annular ring of bone spicule pigmentation just outside of the arcades with some underlying and adjacent retinal pigment epithelium depigmentation typical of a pericentral type of retinitis pigmentosa. Optical coherence tomography (OCT) scans of the maculae show a bilateral loss of photoreceptor outer segments outside of the central macula. On the right there is cystoid macular oedema (CMO), while on the left there is a thick epiretinal membrane (previous CMO). A full-field electroretinogram showed a pattern of severe retinal dysfunction in a rod–cone pattern, with additional macular dysfunction.

In Fabry disease, cornea verticillata may be absent in milder presentations [29]. In Gaucher disease type 1, ocular involvement is uncommon in late-onset cases; however, neuronopathic forms may have decreased horizontal saccadic eye movement. Retinopathy was observed in cases with MPS I (Case 1), MPS II (Case 2) (Figure 3), and MPS IIIA (Case 3), while Fuchs’ dystrophy and glaucoma were noted in Case 10 with MSD, though these may well be coincidental rather than related to the underlying condition (Table 1) [66].

### 4.9. Summary

Attenuated LSDs present with multisystem involvement that is often subtle, slowly progressive, and diagnostically challenging. Cardiac valvulopathy, sensorineural hearing loss, airway abnormalities, and musculoskeletal complications were common in our cohort, while neurological features varied by subtype. Cognitive function was largely preserved, though selective impairments were noted. Hepatosplenomegaly and cutaneous signs were infrequent. These findings highlight the clinical heterogeneity of LSDs in adulthood (in comparison to their classical counterpart) and underscore the importance of a multidisciplinary/multispecialty diagnostic approach to facilitate timely recognition and intervention.

## 5. Biomarkers

### 5.1. Specific Enzyme Activity

Enzyme activity assays remain the diagnostic cornerstone for LSDs [67]. However, in milder phenotypes, enzyme activity often correlates poorly with clinical severity. Residual enzyme activity is frequently detectable and may exceed conventional deficiency thresholds. A robust genotype–phenotype correlation is lacking across many LSD subtypes. For instance, in MPS IVA, residual N-acetylgalactosamine-6-sulfatase activity >1% is generally associated with milder disease, though numerous phenotypic exceptions are documented [11]. Diagnostic interpretation is further complicated by the presence of pseudodeficiency alleles, which can result in reduced in vitro enzyme activity in the absence of clinical disease, challenging the specificity of enzyme testing [7,68]. In our cohort, residual enzyme activity varied widely among patients and did not consistently correlate with clinical severity or organ involvement. Notably, individuals with similar low enzyme activity exhibited variable degrees of multisystem disease, underscoring the limitations of relying on enzyme assays alone for predicting phenotype. Residual enzyme activity in patients is often not clear when measured in a readily available sample source such as leucocytes, but there is clearer measurable residual activity when measured in fibroblasts [69,70,71].

### 5.2. Primary Storage Metabolites

Biomarkers that directly reflect substrate accumulation due to lysosomal enzyme deficiencies can be measured both in blood and urine. Glucosylsphingosine (Lyso-Gb1), the deacylated derivative of glucosylceramide, has emerged as a highly sensitive and specific biomarker for both diagnosis and monitoring of Gaucher disease, including in milder phenotypes (non-neuronopathic type 1) [72,73]. Blood Lyso-Gb1 levels correlate well with disease burden, including hepatosplenomegaly, skeletal involvement, and haematological abnormalities [74], although enzymatic activity remains the gold standard for diagnosis. In addition, some patients may have relatively low levels of Lyso-Gb1 but develop complications such as bone infarcts. Similarly, globotriaosylsphingosine (Lyso-Gb3), the deacylated, water-soluble analogue of globotriaosylceramide (Gb3), is the major glycolipid that accumulates in Fabry disease. While markedly elevated in classical presentations, Lyso-Gb3 levels may be normal in late-onset or heterozygous female patients [75]. Baseline Lyso-Gb3 levels correlate with the severity of the pathogenic variant and decline in response to therapy, supporting their utility in both diagnosis and longitudinal monitoring [76,77]. In NPB, lysosphingomyelin (Lyso-SM), the deacylated derivative of sphingomyelin, is significantly elevated, including in late-onset phenotypes, correlating with disease burden and showing dramatically reduced concentrations in response to ERT; the same is true of N-palmitoyl-O-phosphocholineserine (PPCS, formerly lysoSM-509) [78]. For Krabbe disease, psychosine (galactosylsphingosine) serves as a specific second-tier diagnostic marker in neonatal screening, sensitive for infantile-onset disease. However, its utility in late-onset or milder forms remains under evaluation [79].

Long-chain, sulfated GAGs (heparan, dermatan, and keratan sulfates) are accumulated in MPS and are excreted in urine, making uGAGs a classic screening biomarker [72]. Thus, uGAGs remain the cornerstone screening test for MPS, yet their reliability is limited in attenuated phenotypes, where false-negative results are not uncommon due to lower levels of GAG accumulation and excretion [80,81]. The attenuation in phenotype is paralleled by less-marked GAG elevation, which may complicate detection. Standard practice involves quantitative measurement by dimethylmethylene blue (DMB) assay, adjusted for age. Yet, normal total uGAG levels do not exclude disease, particularly in milder forms. Consequently, electrophoretic qualitative profiling is essential, as it may reveal abnormal uGAG patterns even when total excretion appears within normal limits [82,83]. Such diagnostic pitfalls complicate early detection and may delay diagnosis in attenuated MPS. However, reliance solely on uGAG quantification may miss subtle phenotypes. For that reason, more sensitive and specific techniques are now used for urinary GAG quantification, particularly for the purpose of newborn screening [84]. Urinary oligosaccharides offer an additional discriminatory tool, elevated in ML II/III and Alpha- and Beta-mannosidosis, thereby facilitating differentiation from other LSDs [85,86]. Sulfated galactocerebrosides (sulfatides), critical for myelin stability, are accumulated in MLD and are excreted in urine, whilst their measurement in dried blood spots is the focus of an international collaboration in neonatal screening [72,87,88,89,90]. In Alpha-mannosidosis, mannose-rich oligosaccharides are characteristically elevated in urine [91]. Nonetheless, interpretation of these markers requires clinical correlation, especially in late-onset cases where biomarker levels may be less pronounced [42]. Glucotetrasaccharide (Glc4), a tetrasaccharide byproduct of glycogen degradation, accumulates in Pompe disease. Glc4 levels correlate with muscle glycogen burden, disease activity, and progression. It is a useful test for diagnosing PD; however, its utility in late-onset Pompe disease requires further investigation [92,93].

### 5.3. Biomarkers of Macrophage Activation

Macrophage-derived biomarkers, also referred to as “storage-cell markers”, reflect macrophage activation secondary to substrate accumulation and are indicative of lysosomal disease burden. These biomarkers are particularly relevant in disorders characterised by the accumulation of unmetabolized lipids or glycoproteins within tissue macrophages. Key markers include chitotriosidase, pulmonary and activation-regulated chemokine (CCL18/PARC), and macrophage inflammatory proteins (MIPs) [72]. Chitotriosidase, a chitinase secreted by activated macrophages (especially lipid-laden Gaucher cells), is markedly elevated in Gaucher disease and variably elevated in other lysosomal storage disorders, including but not limited to Niemann–Pick disease type A, B, and C (ASMD, NPC), MLD, Krabbe disease, galactosialidosis, and LALD. Chitotriosidase activity is reported to be increased in many non-lysosomal disease states [94]. Its plasma levels correlate with systemic disease burden and response to enzyme replacement therapy (ERT), making it a widely used surrogate marker for Gaucher disease management [95]. However, its utility is limited by its low specificity and sensitivity, and it is inherently flawed by genetic polymorphisms affecting expression of the CH1T1 gene; approximately 5% of the general population have null activity of chitotriosidase [96]. CCL18 (PARC) is another macrophage-secreted chemokine that is elevated in response to lysosomal substrate accumulation. It is particularly useful in chitotriosidase-deficient individuals and serves as an alternative biomarker for disease monitoring in Gaucher disease. CCL18 levels have been shown to decrease in response to effective ERT and are increasingly used in conjunction with Lyso-Gb1 for treatment monitoring [97]. MIP-1, particularly MIP-1β, is a pro-inflammatory chemokine released by activated macrophages. Elevated MIP-1β levels have been linked with skeletal complications in Gaucher disease and correlate with bone involvement and marrow infiltration, thereby serving as potential biomarkers for monitoring osseous disease burden [98].

### 5.4. Downstream and Secondary Biomarkers

In addition to substrate-specific and macrophage-derived biomarkers, a range of downstream indicators of tissue inflammation, organ damage, and secondary metabolic disruption can be observed in LSDs. Patients with ASMD develop dyslipidaemia primarily due to lysosomal accumulation of sphingomyelin within cells of the reticuloendothelial system disrupting lipid trafficking and secondarily affecting the lipoprotein metabolism. Many ASMD patients demonstrate an atherogenic lipid profile associated with accelerated atherosclerosis [99,100]. In NPC, impaired intracellular cholesterol trafficking leads to cholesterol accumulation and increased oxidative stress, producing oxysterols (indirect plasma biomarkers such as cholestane-3β,5α,6β-triol and 7-ketocholesterol). While not disease-specific, these oxysterols are replaced with lysosphingolipids (PPCS (LSM-509) and bile acid species), which serve as indirect plasma biomarkers supporting diagnosis and disease monitoring [72,101]. In LAL deficiency, adult-onset forms typically present with dyslipidaemia, characterised by elevated low-density lipoprotein (LDL) and reduced high density lipoprotein (HDL) cholesterol and mildly increased hepatic transaminases [43].

Cardiac and renal biomarkers, such as troponin, creatinine kinase, NT-proBNP, albuminuria, and estimated glomerular filtration rate (eGFR), can assist in tracking organ-specific disease involvement [102]. Novel markers, including neurofilament light chain (NfL) and oxysterols, are under investigation for NPC and GM2 gangliosidosis, with potential relevance to disease monitoring and progression, particularly in adult-onset phenotypes [103]. In adult forms of GM1 and GM2 gangliosidosis, plasma ganglioside accumulation and aspartate transaminase (AST) levels may loosely correlate with disease progression, but inter-individual variability remains high [104]. In adult-onset leukodystrophies, such as late-onset MLD, elevated cerebrospinal fluid (CSF) protein may indicate underlying neuroaxonal damage [24].

### 5.5. Summary

Biochemical biomarkers remain central to the diagnosis and monitoring of LSDs; however, attenuated phenotypes pose significant diagnostic challenges. While enzyme assays and genetics remain diagnostic cornerstones in LSDs, other plasma and urine biomarkers can provide valuable tools for screening, monitoring therapeutic response, and tracking disease progression, especially in neurologic and skeletal manifestations. Continued refinement through omics and clinical trials will enhance their role in precision medicine for LSDs. Second-line or adjunctive biomarkers are increasingly employed to improve diagnostic specificity and support phenotypic stratification.

## 6. Molecular Analysis

Attenuated phenotypes are frequently associated with compound heterozygosity involving at least one missense, intronic, or hypomorphic variant that permits residual enzymatic activity [105,106,107]. In contrast, classical early-onset forms are more often linked to homozygous null (e.g., splicing, frameshift, and nonsense) variants or large deletions that result in near-complete enzyme deficiency [106,108,109]. However, the pathogenicity of a missense variant also depends more on its structural and functional impact than its position in the sequence. Harmful variants often affect key amino acids involved in folding or domain interfaces, amino acids in the active site or in the hydrophobic core, while tolerated variants usually occur on the surface or involve minimal structural changes [106,110,111]. For instance, one patient was homozygous for the missense variant c.866A>G; p.(Tyr289Cys), yet displayed a late-onset slowly progressive phenotype of MSD [66]. Furthermore, phenotypic variability may be influenced by modifier genes, epigenetics, or environmental factors [112].

In our cohort, molecular data were available for all 10 patients (Table 2). Eight patients were compound heterozygotes, carrying at least one missense or presumed hypomorphic allele, while Case 6 with MPS VI carried p.(Tyr210Cys) and p.(Trp312Cys), both missense changes. Similarly, patients with Alpha- and Beta-mannosidosis (Case 7 & 8) carried frameshift or stop-gain mutations in trans with milder missense variants [75]. All patients had at least one hypomorphic allele permitting residual activity. One patient was homozygous for a presumed hypomorphic variant in SUMF1, displaying a late-onset slowly progressive phenotype of MSD. Phenotypic variability may additionally be influenced by modifier genes, epigenetics, or environmental factors [83]. Case 7 (Apha-mannosidosis) was compound heterozygous for a frameshift variant and a missense variant. Correspondingly, patients with Beta-mannosidosis carried frameshift or stop-gain mutations in trans with milder missense variants [86].

According to the previous studies, the most common variants in patients with the attenuated (MPS-IH/S and MPS-IS) disease phenotypes were missense (71.8%) followed by nonsense (20.6%), small deletions with no frameshift (3.2%), and splice-site (2.8%), while 97.5% of patients with an attenuated disease phenotype had at least one missense variant [105]. Missense variants altering amino acid residues on the surface of the enzyme have been identified as variants associated with the attenuated disease phenotypes [106,111]. Similarly, there is a marked heterogeneity in the genetic variants detected in individuals with a milder MPS II phenotype. The reported genetic variants are predominantly missense variants, while splice-site variants and nonsense variants have also been reported [108,113,114,115,116,117,118,119,120,121,122,123]. Interestingly, a missense variant, p.Ala77Asp, has been reported in a family lineage with a very attenuated MPS II phenotype, and 2/16 affected members lived beyond 70 years, while one had a mild learning disability [19]. Furthermore, genotypes detected in individuals with late-onset and attenuated MPS IIIA mostly include missense variants [107,124,125,126,127,128].

Missense mutations represented the most frequently observed type of molecular alteration in this cohort. Structural variants or large deletions were not identified. Larger datasets have also demonstrated that various disorders are often associated with residual enzyme function, typically enabled by the presence of at least one mild allele [29,31]. Furthermore, the high frequency of novel or unclassified variants in adult-onset cases underscores the importance of functional validation and genotype–phenotype correlation in achieving accurate diagnosis [5,7]. In silico tools and homology modelling have been employed to study genotype–phenotype correlations in LSDs), aiding in the identification of novel mutations and understanding their pathogenic mechanisms [129,130], Additionally, predictive models using protein sequence and structural data have enhanced insights into disease processes in conditions like Fabry disease [131].

## 7. Discussion

The inconsistent and evolving nomenclature surrounding LSDs, particularly regarding attenuated, late-onset, variant, or non-classical phenotypes, poses a significant challenge in both clinical practice and research. The lack of standardised terminology across clinical and scientific domains generates confusion among healthcare professionals, patients, and researchers, impairing effective communication and collaboration. This variability not only hampers the development of robust clinical guidelines but also complicates patient identification, phenotype stratification, and recruitment in clinical trials. Consequently, it undermines therapeutic decision-making and interpretation of diagnostic and treatment outcomes [5,12,29,132]. Establishing a harmonised nomenclature, alongside consensus diagnostic criteria, is therefore imperative to improving diagnostic accuracy, facilitating multicentre collaboration, and streamlining patient care pathways. To support this aim, a comparative summary of early-onset versus later-onset features in each disorder represented in our cohort is provided in Table 3. This overview highlights how milder forms may deviate significantly from classical presentations and is intended to assist clinicians in recognising subtle, organ-limited, or atypical features that may otherwise be overlooked.

Within the LSD spectrum, MPS is notable for its marked phenotypic heterogeneity. In attenuated forms of MPS I (Scheie syndrome) and MPS II, adult-onset joint stiffness or arthropathy often predominate, frequently mimicking common rheumatological conditions [133]. Classical dysmorphic features and airway abnormalities may be minimal or absent at presentation, complicating early recognition. Airway involvement may develop progressively, including a bulky tongue, supraglottic changes, neck movement restriction, curvature of the trachea, and flattening of the trachea. OSA with dysmorphic features in early childhood needing adenotonsillectomy should raise suspicion of LSD. Hepatosplenomegaly is generally absent, and hearing loss tends to be mild. Early-onset sensorineural hearing loss has been observed in our cohort; this should prompt suspicion for LSD. Importantly, ophthalmological manifestations may frequently offer critical diagnostic clues in attenuated MPS and should prompt further metabolic evaluation when encountered in conjunction with musculoskeletal symptoms [59,133]. Additionally, behavioural and neuropsychiatric symptoms, occasionally predominant in milder MPS and ML, can obscure the underlying diagnosis, especially when they precede somatic signs [32,34].

Several other LSDs, including Gaucher disease, ASMD, and Fabry disease, exemplify the diagnostic challenges posed by attenuated presentations. Although Gaucher disease classically presents in childhood, it can remain undiagnosed until adulthood, with thrombocytopenia often being the presenting feature (see the Supplementary Materials for an analysis of unpublished data from our centre on attenuated Gaucher disease). Notably, pathogenic variants in the GBA gene coding for glucocerebrosidase have been implicated in increased susceptibility to parkinsonism, occasionally constituting the presenting phenotype in carriers [40,41,43,134], who have a similar risk of parkinsonism as patients with Gaucher disease. Similarly, Fabry disease frequently escapes early diagnosis, especially when manifesting as renal-limited disease or isolated hypertrophic cardiomyopathy, often resulting in misdiagnosis and delayed treatment [29]. These diagnostic difficulties highlight the need for increased clinical awareness across specialties, and for the early integration of enzymatic and molecular testing in unexplained systemic finding evaluation. Given the availability of disease-modifying therapies, timely diagnosis is critical not only for initiating appropriate management but also for improving understanding of the natural history and optimising long-term outcomes [135]. Diagnostic advancements, particularly in specialised imaging modalities such as cardiac magnetic resonance imaging (MRI), have improved the detection of subtle myocardial involvement that may be overlooked by conventional echocardiography. These improvements have contributed to the increasing recognition of milder/late-onset forms presenting predominantly with cardiac manifestations [136].

Diagnostic complexity in attenuated LSDs is often compounded by frequent isolated organ involvement. Multisystemic symptoms, when present, tend to be nonspecific and asynchronous, resulting in fragmented care and delayed recognition. For instance, isolated ocular involvement may in fact prompt ophthalmological evaluation, leading to LSD diagnosis [21,137]. Although the exact pathophysiology remains incompletely understood, organ-specific manifestations likely reflect tissue-specific enzyme expression, differential substrate accumulation, and local microenvironmental factors influencing pathogenesis [138]. Moreover, certain pathogenic variants may result in residual enzyme activity that is sufficient to prevent systemic involvement, while still allowing substrate accumulation in vulnerable tissues, such as the cornea, myocardium, or central nervous system. This highlights the importance of recognising tissue-specific phenotypes for accurate diagnosis and personalised management [3,139].

Traditional screening approaches, such as uGAG quantification, exhibit reduced sensitivity in detecting attenuated MPS phenotypes and may yield false-negative results. Consequently, enzymatic assays and molecular genetic testing remain essential components of the diagnostic pathway for LSDs. The widespread adoption of NGS panels has significantly enhanced the detection of atypical and late-onset cases, facilitating broader genotype–phenotype correlation and enabling cascade testing within affected families. Importantly, some cases diagnosed by the genetic testing have abnormal biochemical tests, but they have never been investigated. The variable concordance between genotype and phenotype remains a recognised limitation [5,7,80,82]. Molecular testing may reveal compound heterozygosity or identify only a single pathogenic variant, findings that may reflect current technical constraints highlighting the importance of not relying solely on enzymatic activity studies. The incorporation of second-line biomarkers, such as psychosine in Krabbe disease and lysosphingolipids in Gaucher, ASMD, and Fabry diseases, has further enhanced diagnostic precision and supports longitudinal disease monitoring. Nonetheless, these tools require ongoing validation, particularly in adult-onset presentations [73,75,79]. Additionally, functional assessment of pathogenicity in affected tissues or in vitro assays remains a valuable supplement to diagnostic strategy (i.e., cultured fibroblasts) [140].

Therapeutic decision-making in attenuated LSDs remains particularly challenging due to limited evidence regarding optimal timing and efficacy of treatment in mild or slowly progressive disease. The growing population of diagnosed adults and elderly patients introduces complexities due to age-related comorbidities, complicating clinical assessment and management. Upon diagnosis, a comprehensive multidisciplinary evaluation, including cardiac imaging, ENT, respiratory function testing, neurocognitive evaluation, and abdominal imaging, is essential in order to tailor care. In addition, a detailed family history may reveal subtle manifestations in relatives, offering further diagnostic insights and enabling early identification of at-risk individuals [6,12]. Importantly, maintaining a high index of suspicion is critical, as conventional biomarkers may be normal or borderline in attenuated disease. Increased awareness among primary care physicians and specialists is vital to reduce diagnostic delays and improve outcomes through timely intervention [7,12,13,29,33].

## 8. Conclusions

Despite significant advances in molecular diagnostics, substantial challenges persist in the early recognition and comprehensive characterisation of attenuated LSDs. The clinical heterogeneity and often subtle, organ-specific manifestations contribute to frequent diagnostic delays and underdiagnosis, particularly in adult populations. Our findings underscore the critical role of awareness of LSDs, integrating detailed clinical assessment with enzymatic, molecular, and emerging biomarker analyses to improve diagnostic accuracy. Moreover, inconsistent nomenclature and limited genotype–phenotype correlations hinder standardised care and impede clinical trial enrolment. Looking ahead, there is an urgent need for prospective natural history studies, large-scale genotype–phenotype correlation efforts, and robust clinical registries to delineate disease trajectories and optimise therapeutic strategies in this under-recognised patient cohort. Enhanced multidisciplinary collaboration and heightened clinical vigilance across specialties are essential to facilitate timely diagnosis, guide personalised management, and ultimately improve outcomes for patients with attenuated LSDs.

## Figures and Tables

**Table 1:** Clinical summary of attenuated lysosomal storage disease (LSD) cases.

LSD	Age at Onset of Symptoms	Age at Diagnosis/Gender	Ht (cm)	Wt (kg)	BMI (kg/m^2^)	Enzyme Activity	Cardiovascular	Respiratory/ENT/Airways	Liver/Spleen/ Hernia	Orthopaedics	Neurology/ CNS/ID	Ophthalmology
MPS I (Case 1)	40s—vision impairment, gradual deterioration over time	56/M	177	84	26.9	Alpha- iduronidase 0.3 nmol/mg/h (13.0–111.0)	ECG: LVH on voltage criteria	Chest infections + Right sensorineural deafness ++ PFTs N	USS: N	Changes in lumbar spine: ++	No ID	RP ++ (pericentral subtype) Corneas clear
MPS II (Case 2)	50s—hernia and AF	52/M	181	98	29.8	Iduronate sulfatase 2 nmol/mL/4 h (494–1113)	ECG:AF ECHO: LVH, mild MR CMR: Focal myocardial fibrosis	ENT: Mild narrowing of right main bronchus Bilateral sensory neural hearing loss ++ PFTs N	No hepatosplenomegaly Left inguinal hernia repair in infancy and repeated in his 50s	Retrolisthesis of L2–L3 and L3 on L4 + Small central disc protrusion at C3/C4 + Multilevel loss of disc space height + Degenerative changes in right MTPJ +	No ID Carpal tunnel syndrome + Ulnar neuropathies + Bilateral C4 nerve root compression +	RP +
MPS IIIA (Case 3)	ID and hearing impairment since childhood	40/M *	179	73	22.8	Sulfamidase 0.2 nmol/mg/17 h (3.2–20)	ECG: RBBB, LVH ECHO: N	Chest infections + Mild hearing impairment + Ear infections and grommets + adenoid removed in childhood	Hepatomegaly +	CTS +	ID + Lived independently until 42 yrs, communicative Sudden neurocognitive decline after that: Disturbed sleep, acute psychosis	RP ++
MPS IVA (Case 4)	Childhood—skeletal deformities and pain	8/M *	133	46	26	Galactose 6 sulfatase 0.3 nmol/20 h/mg (4–11)	ECG: Short PR ECHO: N	Conductive hearing impairment + ENT: Contracted nasopharynx, restricted neck extension, high anterior larynx, bulky supraglottis PFTs N	No	Thoracolumbar, spinal and chest deformities +++ Hip fracture Left tibia fracture Foot drop in the right.	No ID He is independent Bilateral weakness, not being able to walk 10 m in 2024	None
MPS IVA (Case 5)	Adolescence—bone deformities and pain	33/M *	160	93	36.4	Galactose 6 sulfatase 0.3 nmol/20 h/mg (4–11)	ECG: SR 101/min normal ECHO: N	ENT: Large tongue, small maxilla, large mandible, poor dentition, high anterior larynx, bulky supraglottis, short neck, restricted neck extension, short cervical spine, curved lower trachea, mild flattening of lower trachea	Splenomegaly and fatty liver +	Dysplastic hips and severe bilateral OA; bilateral OA changes in both knees; orthopaedic intervention of left hip at 11 y/o 2025: Mild degenerative changes in spine	No neurological impairments	None
MPS VI (Case 6)	Childhood—hip replacement and hearing impairment	46/F	147	54	25.2	Arylsulfatase B 0.6 nmol/mg/h (7–108)	ECG: N ECHO: Severe AS, mitral thickening, mild mitral regurgitation	ENT: Adenoidectomy at 29 yr Crowded oropharynx, high anterior larynx, bulky epiglottis Short cervical spine, restricted neck extension Sensorineural hearing	None	Right hip replacement Maxillary slightly hypoplastic	Poor memory, no other cognitive impairment; mild syrinx at C7 no gross cord signal abnormalities; possible Chiari I malformation.	None
Alpha -mannosidosis (Case 7)	Childhood—ID and hearing impairment since age of 3	31/F *	148	68	30.6	2 nmol/h/h (20–100)	ECG: N ECHO: Mild AS, mitral thickening	ENT: Bulky tonsils almost occluding oropharynx; supraglottis moderately bulky Small epiglottis High anterior larynx, mild flattening and curved trachea; restricted neck extension Sensorineural hearing problems	None	CTS: ++ L hip AO Bilateral Genu valgum	Moderate ID Speech impairment; broad-based ataxic gait pattern	Likely retinal changes
Beta -mannosidosis (Case 8)	Childhood—ID	30/F *	163	64	24.1	Leucocyte beta-mannosidase 2 nmol/mL/h (150–1500)	ECG: Short PR interval, RBBB ECHO: Moderate aortic and tricuspid regurgitation	Coarse facial features + Sensorineural hearing loss ++	None	DEXA: Normal bone density	Moderate ID Tremor in upper limbs; Ataxia MRI: Normal	N/A
Krabbe disease (Case 9)	30s—tendency to fall 55—difficulty walking	55/M *	171	95 kg	32.1	GALC 0.03 nmol/mg/h [0.4–4]	ECG: N	N/A	Umbilical hernia	No	Acroparesthesia Ataxia Brisk reflexes with bilateral clonus; Babinsky + bilateral. no saddle paraesthesia or anal sphincter dysfunction MRI: Signal changes in corticospinal tracts and symmetrical parietal white matter high signal intensities on FLAIR images NCS: Consistent with chronic L5 radiculopathy	None
Multiple sulfatases (Case 10)	40s—progression of vision impairment	52/F *	165	73	25	Several Arylsulfatase Alpha reduced ++ Heparin sulfamidase reduced +	ECG: ECHO: LVH, grade 1 diastolic dysfunction	None	Mild fatty liver and gallstones	None	No ID MRI: Non-specific white matter changes	Bilateral retinal dystrophy; Fuch’s corneal endothelial dystrophy Glaucoma

Note: Asterisks (*) indicate cases that have been previously included in published manuscripts or abstracts. Abbreviations: Lysosomal Storage Disease (LSD), Height (Ht), Weight (Wt), Body Mass Index (BMI), Mucopolysaccharidosis (MPS), Pulmonary Function Tests (PFTs), Ultrasound Scan (USS), Electrocardiogram (ECG), Echocardiogram (ECHO), Cardiac Magnetic Resonance Imaging (CMR), Sinus Rhythm (SR), Atrial Fibrillation (AF), Aortic Stenosis (AS), Left Ventricular Hypertrophy (LVH), Mitral Regurgitation (MR), Metacarpal–Phalangeal Joint (MTPJ), Right Bundle Branch Block (RBBB), Osteoarthritis (OA), Intellectual Disability (ID), Carpal Tunnel Syndrome (CTS), Nerve Conduction Study (NCS), Magnetic Resonance Imaging (MRI), Central Nervous System (CNS), Fluid-Attenuated Inversion Recovery (FLAIR), Ear, Nose, and Throat (ENT), Retinitis Pigmentosa (RP). Present (+), present significantly (++), present in abundance (+++), normal (N).

**Table 2:** Genetic variants identified in patients (all Caucasian) with lysosomal storage disorders (LSDs): Variant classifications are based on the American College of Medical Genetics and Genomics/Association for Molecular Pathology (ACMG/AMP) 2015 guidelines and are interpreted using publicly available databases such as the Clinical Variant Database (ClinVar) and Variant Interpretation Platform (VarSome). Protein changes are described using standard Human Genome Variation Society (HGVS) nomenclature. The mutation type refers to the molecular nature of the variant, such as missense (amino acid substitution), nonsense (premature stop codon), or frameshift (insertions or deletions causing a shift in the reading frame). Inheritance patterns are described as homozygous (same mutation on both alleles), compound heterozygous (two different mutations, one on each allele), or heterozygous (a single detected variant). Abbreviations include the following: Alpha-L-iduronidase (*IDUA*), Iduronate 2-sulfatase (*IDS*), N-sulfoglucosamine sulfohydrolase (*SGSH*), N-acetylgalactosamine-6-sulfatase (*GALNS*), Arylsulfatase B (*ARSB*), Mannosidase alpha class 2B member 1 (*MAN2B1*), Galactosylceramidase (*GALC*), Mannosidase beta (*MANBA*), and Sulfatase modifying factor 1 (*SUMF1*).

LSD	Gene	Molecular	Type	Protein Change	Classification	Inheritance Pattern
MPS I (Case 1)	*IDUA*	c.794G>A p and c.1205 G>A	Missense and Missense	p.(Gly265Asp) and p.(Trp402Ter)	Pathogenic and Pathogenic	Compound Heterozygous
MPS II (Case 2)	*IDS*	c.817C>T	Missense	p.(Arg273Trp)	Pathogenic	Homozygous
MPS IIIA (Case 3)	*SGSH*	c.1063G>A and c.220C>T	Missense and Missense	p.(Glu355Lys) and p.(Arg74Cys)	Pathogenic and Pathogenic	Compound Heterozygous
MPS IVA (Case 4)	*GALNS*	c.331C>T and N/A	Missense	p.(Gln111Ter) and p.(Ala241Ala)	Pathogenic Likely Pathogenic	Compound Heterozygous
MPS IVA (Case 5)	*GALNS*	c.604del and c.143T>G	Stop Codon and Missense	p.(Glu202LysfsTer117) and p.(Val48Gly)	Pathogenic and Likely Pathogenic	Compound Heterozygous
MPS VI (Case 6)	*ARSB*	c.629A>G and c.936G>T	Missense and Missense	p.(Tyr210Cys) and p.(Trp312Cys)	Likely Pathogenic and Likely Pathogenic	Compound Heterozygous
Alpha -mannosidosis (Case 7)	*MAN2B1*	c.1388_1389del and c.2426T>C	Frameshift and Missense	p.(Arg463Profs53) and p.(Leu809Pro)	Pathogenic and Likely Pathogenic	Compound Heterozygous
Beta -mannosidosis (Case 8)	*MANBA*	c.1452_1453del and c.1753C>T	Frameshift and Stop Codon	p.(Tyr485CysfsTer27) and p.(Arg585Ter)	Pathogenic and Pathogenic	Compound Heterozygous
Krabbe disease (Case 9)	*GALC*	c.326C>T and c.391T>C	Missense and Missense	p.(Thr109Ile) and p.(Trp131Arg)	Pathogenic and Pathogenic	Compound Heterozygous
Multiple sulfatases (Case 10)	*SUMF1*	c.866A>G	Missense	p.(Tyr289Cys)	Likely Pathogenic	Homozygous

**Table 3:** A comparison of early-onset (classical) and later-onset (attenuated) features across lysosomal storage disorders (LSDs) represented in the study cohort: This table summarises the classical early-onset features typically associated with each LSD, contrasted with the later-onset manifestations observed in the adult patients within our cohort. The comparison aims to illustrate how clinical presentation can evolve across the phenotypic spectrum, particularly in milder forms that may present with isolated or subtle organ involvement. This overview is intended as a practical guide to support recognition of atypical presentations by non-specialists.

LSD	Early-Onset (Classical) Presentation	Late-Onset (Attenuated) Presentation
MPS I	Coarse facial features, developmental delay, skeletal dysplasia, corneal clouding	Isolated retinal dystrophy, mild LVH, clear corneas, normal cognition
MPS II	Hepatosplenomegaly, cognitive decline, skeletal deformities, coarse facies	Cardiac fibrosis, atrial fibrillation, mild airway and joint changes
MPS IIIA	Severe neurodegeneration, behavioural disturbance, early cognitive loss	Late-onset psychosis, progressive cognitive decline after stable adult life
MPS IVA	Skeletal dysplasia, growth delay, short stature, joint stiffness	Orthopaedic issues, curved trachea, ENT abnormalities, preserved intellect
MPS VI	Joint contractures, coarse facial features, airway abnormalities, hepatosplenomegaly	Severe aortic stenosis, sensorineural hearing loss, Chiari I malformation
Alpha-mannosidosis	Intellectual disability, recurrent infections, hearing loss, skeletal abnormalities	Moderate intellectual disability, speech impairment, broad-based gait, airway abnormalities
Beta-mannosidosis	Intellectual disability, coarse features, hypotonia, seizures	Tremor, ataxia, moderate intellectual disability
Krabbe Disease	Developmental regression, irritability, spasticity, early death	Adult-onset spastic paraparesis, white matter changes
Multiple Sulfatase Deficiency	Developmental delay, ichthyosis, multisystem involvement	Retinal dystrophy, glaucoma, nonspecific brain imaging findings

## Data Availability

No new data were created or analysed in this study. Data sharing is not applicable to this article.

## Supplementary Materials

The following supporting information can be downloaded at: https://www.mdpi.com/article/10.3390/genes16080915/s1, Data: Clinical Characteristics of Adult-Diagnosed Versus Childhood-Diagnosed Gaucher Disease Type 1 Patients at a Single UK Centre.

## Abbreviations

The following abbreviations are used in this manuscript:

ARSB	arylsulfatase B
ASMD	acid sphingomyelinase deficiency
BMI	body mass index
CMR	cardiac magnetic resonance imaging
CNS	central nervous system
CTS	carpal tunnel syndrome
ECG	electrocardiogram
ECHO	echocardiogram
EF	ejection fraction
FLAIR	fluid-attenuated inversion recovery
GAGs	glycosaminoglycans
GALNS	N-acetylgalactosamine-6-sulfatase
GALC	galactosylceramidase
Ht	height
ID	intellectual disability
IDUA	alpha-L-iduronidase
IDS	iduronate 2-sulfatase
LALD	lysosomal acid lipase deficiency
LSD	lysosomal storage disease
LVH	left ventricular hypertrophy
MANBA	mannosidase beta
MAN2B1	mannosidase alpha class 2B member 1
MLD	metachromatic luekodystrophy
MPS	mucopolysaccharidosis
MRI	magnetic resonance imaging
NCS	nerve conduction studies
NPC	Niemann–Pick C
OSA	obstructive sleep apnoea
PFTs	pulmonary function test
SMPD1	sphingomyelin phosphodiesterase 1
SUMF1	sulfatase modifying factor 1
RBBB	right bundle branch block
RP	retinitis pigmentosa
SHSH	N-sulfoglucosamine sulfohydrolase
VUS	variant of unknown significance
USS	ultrasound
Wt	weight
