# Supplementary material for: Genetic Insights and Diagnostic Challenges in Highly Attenuated Lysosomal Storage Disorders
Source: Genes (Basel). 2025 Jul 30;16(8):915. doi: 10.3390/genes16080915 (PMC12385809; doi:10.3390/genes16080915)
Supplement: Supplementary file 1 [file genes-16-00915-s001.zip › genes-3769918-supplementary.pdf]

Supplementary Data

Clinical Characteristics of Adult-Diagnosed Versus Childhood-Diagnosed Gaucher Disease Type 1 Patients at a Single UK Centre

A retrospective analysis of 37 adult patients with Gaucher disease type 1 managed at a single tertiary centre identified 18 individuals who were diagnosed in adulthood (>18 years). The cohort comprised individuals of White British/European (78%), Ashkenazi Jewish (11%), and South Asian (11%) descent. All 18 patients diagnosed in adulthood were initially assessed by a haematologist and referred to the metabolic/genetics clinic with suspected Gaucher. The primary referral triggers included thrombocytopenia and fatigue (3), avascular necrosis of the femoral neck with concurrent thrombocytopenia and osteoporosis (3), and incidental splenomegaly identified during investigations for unrelated concerns (2). One patient underwent splenectomy in her 40s for unexplained thrombocytopenia prior to a diagnosis of Gaucher and subsequently developed mild pulmonary involvement in her 70s, despite treatment with substrate reduction therapy (SRT). Another patient, with long-standing osteoporosis and primary hypothyroidism, was only diagnosed following a traumatic pubic bone fracture in her 50s; her condition later progressed to include monoclonal gammopathy of undetermined significance (MGUS) in her 60s. The adult-diagnosed subgroup commonly presented with thrombocytopenia, fatigue, or incidental splenomegaly, often resulting in delayed diagnosis. The median age at diagnosis was 32 years (range: 21–58), with a median platelet count of 128 × 10^9/L (IQR: 102–145), reflecting mild thrombocytopenia. Despite later initiation of treatment, patients diagnosed in adulthood achieved bone density and haematological outcomes comparable to those diagnosed in childhood. Enzyme activity levels at diagnosis were slightly higher in the adult-diagnosed group, consistent with a milder disease phenotype. The most frequent variants identified were c.1226A>G (p.Asn409Ser) and c.1448T>C (p.Leu483Pro), typically occurring in compound heterozygosity with other missense mutations. SRT, particularly eliglustat, was the preferred treatment modality in this group.

These findings highlight the need to consider Gaucher in adults presenting with unexplained thrombocytopenia, bone pathology, or splenomegaly, and demonstrate that timely intervention can yield clinical benefit even in milder, late-onset cases. Key findings are summarised in the accompanying supplementary table.

**Feature**	**Childhood-Diagnosed**	**Adult-Diagnosed**
Number of patients	19	18
Median age at diagnosis (years)	13 (0.3–13)	32 (21–58)
Gender (F/M)	8F/11M	9F/9M
Platelet count at diagnosis (median, IQR)	Not Available	128 × 10^9/L (IQR: 102–145)
Bone disease	14	10
Splenomegaly (on US or MRI)	5	2
Common genotypes	c.1226A>G / c.1399G>C	c.1226A>G / c.1390C>T
Enzyme activity at diagnosis (nmol/mg/hr)	0.37 (0.16–0.7)	0.44 (0.31–1.05)
Primary therapy (ERT/SRT)	8/11	4/14

Supplemental table: Key Clinical Findings in Childhood-Onset vs. Adult-Onset Gaucher Patients. Abbreviations: F – Female; M – Male; IQR – Interquartile Range; US – Ultrasound; MRI – Magnetic Resonance Imaging; ERT – Enzyme Replacement Therapy; SRT – Substrate Reduction Therapy.
